# Anti-tumor effects of anti-PD-L1 therapy in an orthotopic bladder tumor model

**DOI:** 10.1186/2051-1426-2-S3-P101

**Published:** 2014-11-06

**Authors:** Amanda Lyon, Jonathan Fallon, Benjamin Boyerinas, Robert Schmitz, Kenneth W Hance, Yan Lan, Helen Sabzevari, Kwong Tsang, Jeffrey Schlom, John Greiner

**Affiliations:** 1 CCR, National Cancer Institute, NIH, Bethesda, MD, USA; 2 EMD Serono Research & Development Institute, Inc., Billerica, MA, USA; 3 Laboratory of Tumor Immunology and Biology, CCR, NCI, NIH, Bethesda, MD, USA

## 

Antibodies that block PD-1/PD-L1 interactions have shown efficacy against both lung and skin cancers in early-stage clinical trials, and may also be effective in other tumor types, particularly bladder tumors. PD-L1 expression has been previously reported to correlate with high-grade tumors, a high recurrence rate, and reduced survival rate in patients with bladder cancer. These findings and the high frequency of somatic mutations found in bladder tumors indicate that bladder cancer patients may respond well to anti-PD-L1 therapy. Both murine (MB49, MBT-2) and human (J82, T24, TCCSUP) bladder cancer cell lines constitutively express PD-L1 as determined by flow cytometry. As expected, *in vitro *IFN-γ addition up-regulated PD-L1 expression levels on each of those tumor cells. A human IgG_1 _anti-PD-L1 antibody, MSB0010718C, induced ADCC activity *in vitro *against all three human bladder cancer cell lines following treatment with IFN-γ. In initial *in vivo *murine studies, the growth of s.c. MB49 tumors in syngeneic mice was significantly delayed following three i.p. injections of 400 µg of the MSB0010718C anti-PD-L1 antibody. An orthotopic bladder model consisting of the MB49 cells tagged with luciferase (MB49-luc) was also used to evaluate the antitumor efficacy of the anti-PD-L1 antibody. MB49-luc cells were instilled intravesically (bladder) in B6 mice. Beginning at 7 or 10 days post-instillation, three i.p. injections of the anti-PD-L1 antibody substantially reduced tumor volumes, as determined by intravital imaging, leading to long-term tumor-free survival for 40-60% of the treated mice. While initial immune cell subset depletion studies implicated both CD4+ and CD8+ T cells, continuing efforts will further define the cellular mechanisms responsible for the antitumor effects of the anti-PD-L1 antibody. Taken together, these results suggest that MSB0010718C therapy might be used to activate both innate and adaptive immune mechanisms to treat PD-L1-expressing bladder tumors. Furthermore, the MB49 tumor model can be used to evaluate the combined effects of anti-PD-L1 and other therapeutic agents, particularly ones that induce IFN-γ production and tumor PD-L1 up-regulation. MSB0010718C is currently being evaluated in a Phase I clinical trial (NCT01772004).

